# On the Different Mode of Action of Au(I)/Ag(I)-NHC Bis-Anthracenyl Complexes Towards Selected Target Biomolecules

**DOI:** 10.3390/molecules25225446

**Published:** 2020-11-20

**Authors:** Francesca Binacchi, Federica Guarra, Damiano Cirri, Tiziano Marzo, Alessandro Pratesi, Luigi Messori, Chiara Gabbiani, Tarita Biver

**Affiliations:** 1 Department of Chemistry and Industrial Chemistry, University of Pisa, Via G. Moruzzi 13, 56124 Pisa, Italy; francesca.binacchi@phd.unipi.it (F.B.); guarraf@hotmail.it (F.G.); damiano.cirri@dcci.unipi.it (D.C.); chiara.gabbiani@unipi.it (C.G.); 2 Department of Pharmacy, University of Pisa, Via Bonanno Pisano 6, 56126 Pisa, Italy; tiziano.marzo@unipi.it; 3 Laboratory of Metals in Medicine (MetMed), Department of Chemistry “Ugo Schiff”, University of Florence, Via della Lastruccia 3-13, 50019 Sesto Fiorentino, Italy; luigi.messori@unifi.it

**Keywords:** silver carbene, gold carbene, target selectivity, nucleic acids, quadruplexes, mode of action, protein metalation

## Abstract

Gold and silver N-heterocyclic carbenes (NHCs) are emerging for therapeutic applications. Multiple techniques are here used to unveil the mechanistic details of the binding to different biosubstrates of bis(1-(anthracen-9-ylmethyl)-3-ethylimidazol-2-ylidene) silver chloride [Ag(EIA)_2_]Cl and bis(1-(anthracen-9-ylmethyl)-3-ethylimidazol-2-ylidene) gold chloride [Au(EIA)_2_]Cl. As the biosubstrates, we tested natural double-stranded DNA, synthetic RNA polynucleotides (single-poly(A), double-poly(A)poly(U) and triple-stranded poly(A)2poly(U)), DNA G-quadruplex structures (G4s), and bovine serum albumin (BSA) protein. Absorbance and fluorescence titrations, mass spectrometry together with melting and viscometry tests show significant differences in the binding features between silver and gold compounds. [Au(EIA)_2_]Cl covalently binds BSA. It is here evidenced that the selectivity is high: low affinity and external binding for all polynucleotides and G4s are found. Conversely, in the case of [Ag(EIA)_2_]Cl, the binding to BSA is weak and relies on electrostatic interactions. [Ag(EIA)_2_]Cl strongly/selectively interacts only with double strands by a mechanism where intercalation plays the major role, but groove binding is also operative. The absence of an interaction with triplexes indicates the major role played by the geometrical constraints to drive the binding mode.

## 1. Introduction

Beyond their wide use in catalysis, metal N-heterocyclic carbenes (NHC) are now being extensively investigated for therapeutic applications such as anticancer, antibacterial, antiviral and antiparasitic treatments [1,2]. NHC can coordinate most of the transition metals and confer increased stability in aqueous solution [3]. Moreover, an impressive number of different structures with customized electronic [4] and targeting properties [5] can be prepared with limited synthetic efforts by varying the substituents on the NHC ring. Therefore, it is not surprising that platinum, palladium, ruthenium, rhodium, iridium, copper, silver, and gold NHCs have been tested for their biological properties [1,2,6,7,8]. In this regard, driven by the discovery of the antitumor activity of gold antiarthritic drugs [9], research on gold carbenes mainly focuses on the anticancer properties [10,11]. On the other hand, silver complexes can count on a long history as antimicrobials [12]. Aiming at a more controlled release of silver ions, researchers are trying to prepare silver NHCs boosting the efficacy of silver anti-infective properties [13,14]. Nonetheless, in recent years, the antiproliferative activity of silver carbenes against tumour cells has also been evidenced [15,16,17]. Overall, mechanistic insights concerning gold and silver NHCs have pointed out that the antiproliferative activity against cancer cells is often related to the inhibition of selenol- and thiol-containing enzymes together with antimitochondrial effects [15,16,18,19,20]. However, suitable wingtip substituents can enable increased affinity for DNA: gold and silver NHCs engaging genomic targets are emerging [21]. A benzimidazole-ylidene dicarbenic compound was able to cause DNA fragmentation in glioblastoma cells [22]. These systems may show a multimodal activity: naphtalimide functionalized gold NHCs could both intercalate into the double helix of DNA and exhibit nanomolar TrxR inhibition [23]. A class of cyclometalated gold(III) NHCs was found to be a potent DNA intercalator [24]. Pairwise, for this series of complexes, DNA cleavage and inhibition of Topo-I mediated DNA relaxation were evidenced. In the quest for treatments with limited side effects, the selectivity of complexes towards specific nucleic acid sequences and structures is of utmost importance. In this concern, a panel of gold compounds bearing two caffeine-derived carbene ligands displayed selective binding towards G-quadruplexes over double-stranded DNA [25,26]. Detailed mechanistic studies on the binding modes of these metal complexes to DNA are relatively rare [27,28]. Additionally, it has to be noted that the nucleic acid target cited until now is DNA only: to the best of our knowledge, the binding to RNA polynucleotides is not documented. Binding to serum proteins including albumin is another factor of paramount importance [29,30,31,32]. It has been widely documented that the free and solvent-exposed cysteine residue (i.e., Cys-34) represents the preferential binding site for gold-based coordination compounds [30,33,34,35]. This feature has often been interpreted as a negative factor limiting drug uptake, bioavailability and also responsible for the severe side-effects often associated with the chemotherapy treatments [29,36,37]. However, it has been recently demonstrated that a gold NHC albumin conjugate retains its cytotoxicity and can be employed to deliver the drug into cancer cells [38]. Within this frame, we investigated the interaction of anthracenyl functionalized NHCs with natural double-stranded DNA, telomeric G-quadruplex, synthetic RNA and bovine serum albumin (BSA); this latter was selected as a model protein because of its similarity with the human serum albumin (HSA) and for its easy availability and low cost [39]. Anthracenyl functionalized imidazolylidene complexes of silver and gold were first reported by Rigobello et al. [18] who pointed out a promising antiproliferative activity against cancer cells accompanied by potent inhibition of TrxR. Although fluorescence microscopy showed the accumulation of the complexes in the nucleus, the interaction with DNA was not investigated. Later on, a work by some of us compared the DNA binding features of two anthracenyl benzimidazolylidene monocarbenes of gold and silver [40]. The obtained data revealed that the silver carbene was the most effective double helix DNA stabilizer suggesting an intercalative binding mode for the silver compound, whereas interaction with the groove was hypothesized for the gold carbene. Additional research on bis(1-(9-anthracenylmethyl)-3-ethylimidazol-2-ylidene) silver chloride evidenced a nanomolar TrxR inhibition and an unconventional mode of binding towards the C-terminal TrxR synthetic dodecapeptide endowed of the reactive -Cys-Sec- motif. It has been highlighted that the covalent binding of the -Cys-Sec- amino acid residue with the NHC ligand occurred after silver release [41].

**Figure 1:**
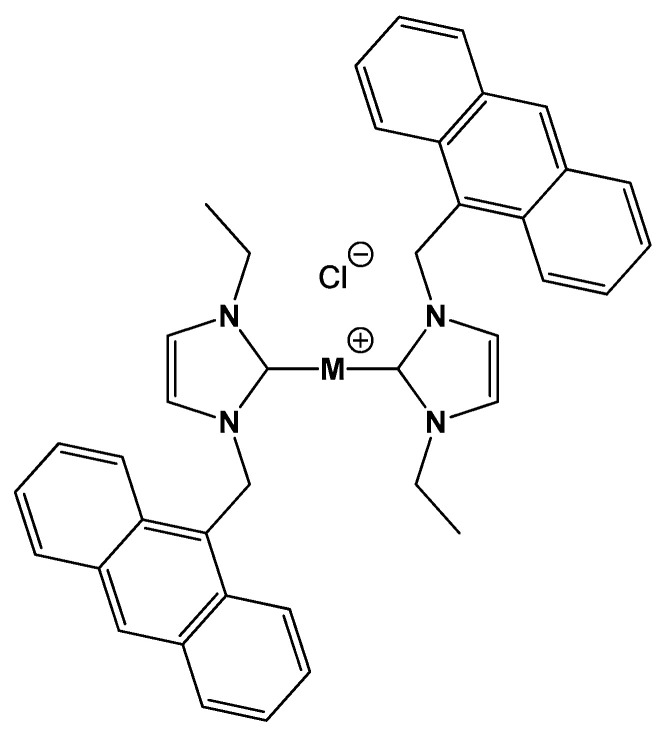
Molecular structure of the M(I)(bis(1-(anthracen-9-ylmethyl)-3-ethylimidazol-2-ylidene) chloride compounds studied: [Ag(EIA)_2_]Cl and [Au(EIA)_2_]Cl.

On this basis, having in mind the relevance of describing interactions at a molecular level for drug optimization, the present research aims at shedding light on the binding features towards different biosubstrates (i.e., calf thymus DNA and RNA polynucleotides, G-quadruplexes, BSA) of the studied Au(I)/Ag(I) bis-anthracenyl carbenes bis(1-(anthracen-9-ylmethyl)-3-ethylimidazol-2-ylidene) silver chloride [Ag(EIA)_2_]Cl and bis(1-(anthracen-9-ylmethyl)-3-ethylimidazol-2-ylidene) gold chloride [Au(EIA)_2_]Cl, schematically depicted in Figure 1. Multi-technique experiments were performed to obtain mechanistic information and highlight a possible selectivity towards a specific biomolecule. A comparison between the different reactivity of the gold and silver carbenes enables us to trace back the observed mechanistic differences to the nature of the metal center.

## 2. Results and Discussion

The synthesis of [Ag(EIA)_2_]Cl compound has already been reported [41]. The [Au(EIA)_2_]Cl compound was synthesized from [Ag(EIA)_2_]Cl using a trans-metalation process described in the experimental section. Note that the compounds are not planar: the exact geometry of [Ag(EIA)_2_]Cl was solved by X-ray analysis yielding a C-Ag bond length of 2.093 (4) Å and a C-Ag-C’ angle of 167.7° [41] (CSD code: PIQBEZ).

### 2.1. Interaction with Natural Double-Stranded DNA

#### 2.1.1. Absorbance and Fluorescence Titrations

**Figure 2:**
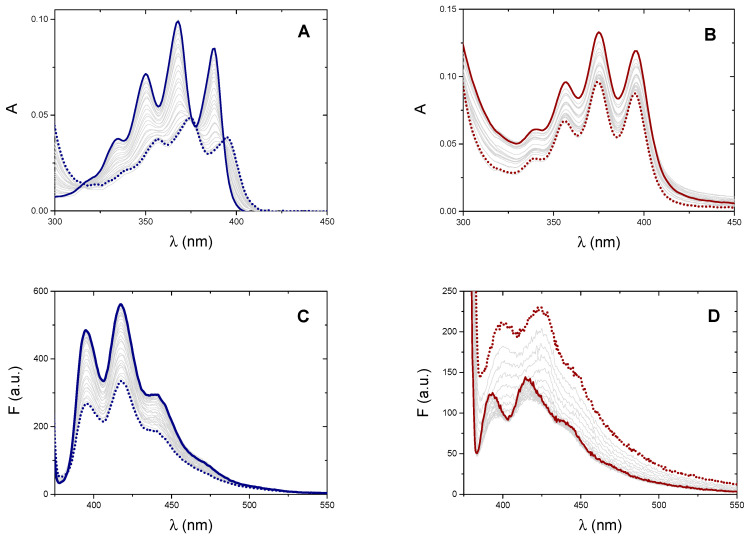
Absorption spectra in the presence of increasing amounts of CT-DNA of (**A**) [Ag(EIA)_2_]^+^ 7.96 × 10^−6^ M alone (full line) and DNA from 0 M to 5.89 × 10^−4^ M (dotted line), (**B**) [Au(EIA)_2_]^+^ 4.34 × 10^−5^ M alone (full line) and DNA, from 0 M to 3.55 × 10^−4^ M (dotted line). Fluorescence spectra in the presence of increasing amounts of CT-DNA of (**C**) [Ag(EIA)_2_]^+^ 7.86 × 10^−8^ M alone (full line) and DNA from 0 M to 7.13 × 10^−5^ M (dotted line), λ_ex_ = 368 nm, (**D**) [Au(EIA)_2_]^+^ 7.71 × 10^−6^ M alone (full line and DNA from 0 M to 8.16 × 10^−4^ M (dotted line), λ_ex_ = 375 nm. NaCl 0.1 M, NaCac 2.5 mM, pH = 7.0, T = 25.0 °C.

To verify the possible interaction between the metal complexes and natural double-stranded DNA (calf thymus DNA, CT-DNA), we performed spectrophotometric titrations by recording the absorption spectra of the two compounds in the presence of increasing amounts of CT-DNA. The polynucleotide is added into the spectrophotometric cuvette containing a known concentration of the tested metal complex (Figure 2). Titrations were carried out at four different temperatures ranging from 15 to 48 °C. Both systems undergo some hypochromic effect but with very different features between silver and gold compounds. For the silver carbene, the spectrum shows a dramatic hypochromic effect, the dye profile significantly changes and undergoes a bathochromic shift of 7 nm; also, an isosbestic point appears at 393 nm (Figure 2A). These results suggest a significant alteration of the electronic levels and may represent the first signal of an intercalative interaction of the silver carbene. Conversely, for the gold complex, the hypochromic effect is much milder and no significant changes nor shift of the absorbance profile is observed (Figure 2B). Therefore, the limited changes in the spectral profile of the gold complex suggest a different type of interaction with double-stranded DNA such as external/groove binding [42] (see also viscometric tests below). Literature data also confirm that, while hypochromic and bathochromic shifts in anthracene vibronic bands indicate the occurrence ofDNA intercalation, hypochromicity in the absence of red shifting is a characteristic of anthracene minor groove interactions [43,44].

If the binding process is described by the simplified apparent reaction below (Equation (1)), whose binding constant is K, the titration curves can be analysed by using Equation (2)

(1) P+D ⇄PD

(2) $$\frac{\Delta A}{{\;C}_{D}}=\;\frac{\Delta \varepsilon \;K\;[P]}{1+K\;[P]}$$

where ΔA = A − (ε_D_C_D_), C_D_ (the total analytical concentration of the dye) = [D] + [PD]; C_P_ (the total analytical concentration of the polynucleotide) = [P] + [PD], Δε = ε_PD_ − ε_D_ is the difference in the molar absorption coefficient of the DNA/dye complex and of the dye alone. Note that Equation (2) is only another way to express the well-known Hildebrand-Benesi equation [45].

**Figure 3:**
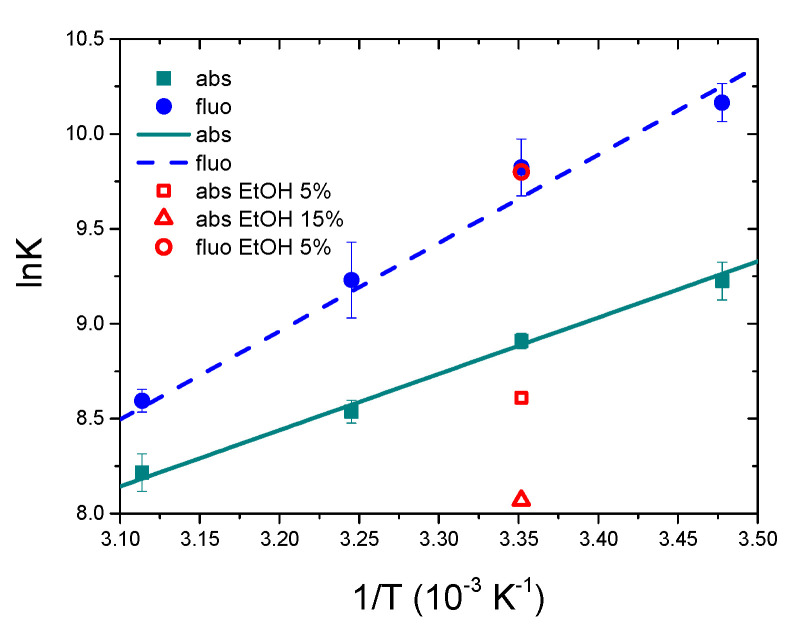
Spectrophotometric and spectrofluorimetric Van’t Hoff plots for the [Ag(EIA)_2_]^+^/CT-DNA system; NaCl 0.1 M, NaCac 2.5 mM, pH = 7.0.

As a first approximation [P] ≈ C_P_ and the first estimate of K is obtained. With this first value of K, [P] = C_P_ − [PD] is calculated, being $\left[\mathrm{PD}\right]=\frac{B-\sqrt{B^{2}-4C_{P}C_{D}}}{2}$ and B = C_P_ + C_D_ + 1/K. This iterative method, at convergence, allowed the calculation of the binding constants for the [Ag(EIA)_2_]^+^/CT-DNA system (Table 1, Figure 3, Supplementary Materials Figure S1A). For the gold carbene (Figure S1B), no convergence occurs and K → +∞, suggesting a quantitative reaction. Note that Scatchard analysis [46] of the data related to the [Ag(EIA)_2_]^+^/CT-DNA system (Figure S2) yields a site size close to one (1.1 ± 0.1). It follows that the simplified equilibrium described by Equation (1), although approximate, represents a valid model for the system studied.

Also, in the case of fluorescence titrations, again a very different spectral behaviour is observed for the two metal complexes (Figure 2C,D). Concerning the silver carbene, increasing amounts of DNA cause a decrease in the emission. Fluorescence data for [Ag(EIA)_2_]^+^ could be analysed using Equation (2), by substituting ΔA and Δε with ΔF = F − (ϕ_D_C_D_) and Δϕ = ϕ_PD_ − ϕ_D_ (Figure S3A). The overall results are shown in Table 1 and plotted in Figure 3. Interestingly, the numerical values obtained by absorbance or fluorescence measurements do not agree with each other and give two different well-defined trends. Linear fitting allowed the calculation of the thermodynamic features for the [Ag(EIA)_2_]^+^/CT-DNA adduct (Table 1): these values both suggest an enthalpy-driven interaction, in line with intercalation [47]. At variance, the spectral variations for the gold compound exhibit a double trend: an initial quenching effect is followed by an increase of the emission with a slight red shift (Figure S3B). The first branch strongly depends on the experimental conditions: it strengthens at higher temperatures (Figure S3C) and fades out at higher salt content (Figure S3D). These features suggest a complex interaction between the gold compound and CT-DNA under dye excess conditions, likely related to some cooperative polynucleotide-induced aggregation effect. Indeed, the effect is observed upon addition of DNA, so it is supposed to be DNA-driven. There are many cases in the literature where the small molecule has a structure, which favours some dye-dye interaction, but the dye is too diluted to aggregate when alone in solution. However, as the first addition of DNA is done in the titration (i.e., under dye excess conditions), the negatively charged DNA will constitute a local aggregation point for the positively charged dye. The dye will locate itself in the vicinity of the helix (we do not know exactly if externally i.e., only with some contact with phosphates, or lying in the grooves) as a consequence of electrostatic attraction. Thus, the local concentration of the dye on the helix surface becomes much higher than the average value in the bulk. So, there, on the helix surface and DNA-driven, aggregation can occur [48,49]. This strongly seems the case here as we see DNA-driven effects which very strongly depend on the ionic strength (high importance of electrostatics as in aggregation processes). Any intercalation contribution is ruled out based on viscosity results (see below). Under these circumstances, the robust study of the second branch, which is likely that connected to the (weak) main binding, was not possible.

To better understand the fact that the numerical values of K_abs_ and K_fluo_ for the [Ag(EIA)_2_]^+^/CT-DNA system do not match, we carried out some titrations in the presence of increasing amounts of ethanol. EtOH has a much lower dielectric constant than water and is known to alter the hydration of polynucleotides [50]. Different hydration of the polynucleotide can guide the system towards a specific binding mode. In particular, changes in hydration levels can affect interactions in the DNA groove to higher extents [51]. Interestingly, absorbance experiments enlighten a decrease of the equilibrium constant value by increasing EtOH%; conversely, K does not change in the fluorescent titrations (red points in Figure 3). Therefore, the K values obtained by the different techniques for the [Ag(EIA)_2_]^+^/CT-DNA system are apparent binding constants that contain the contributions of both groove binding and intercalation. The numerical value is the result of the two contributions, which are differently weighted due to experimental conditions as reactants concentrations or temperature. Different reactant concentrations for absorbance or fluorescence measurements account for the different numerical values obtained at the same temperature by the two techniques. The lowest are concentrations, dye-dye interactions stabilizing groove binding fade out, and intercalation is favoured. The higher the temperature, intercalation (which is a much more exothermic process than groove binding) fades out and groove binding dominates. Temperature dependence is different for K_abs_ and K_fluo_ as they contain a different balance of intercalation and groove binding, which will each depend differently on temperature. It is likely that, at a sufficiently high temperature, K_abs_ ≅ K_fluo_, as the intercalation contribution tends to zero and both numbers will reflect groove binding. Note that the fluorescence parameters obtained here for the silver bis-anthracene are close to what was obtained for the silver mono-anthracenyl compound already studied by some of us (K 25 °C = 1.5 × 10^4^ M^−1^, ΔH = −40 kJ/mol, ΔS = −54 kJ/molK, −TΔS = +16 kJ/mol) [40].

#### 2.1.2. Thermal Denaturation Experiments

**Figure 4:**
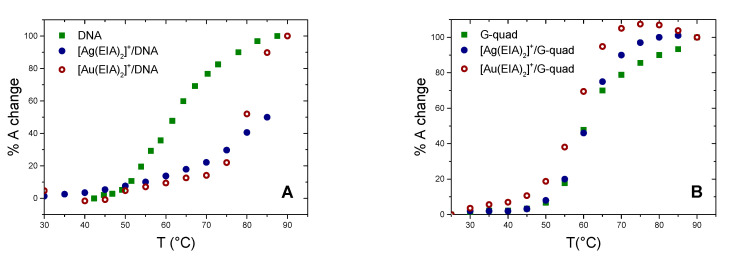
(**A**) Melting plot of DNA alone (C_DNA_ = 2.00 × 10^−5^ M) and DNA with [Ag(EIA)_2_]^+^ or [Au(EIA)_2_]^+^, C_DNA_/C_Ag_ = C_DNA_/C_Au_ = 1, NaCac 2.5 mM, pH = 7.0. (**B**) Melting plot of G4 only (C_G4_ = 4.72 × 10^−7^ M) and in the presence of [Ag(EIA)_2_]^+^ or [Au(EIA)_2_]^+^ added, C_G4_/C_Ag_ = 1.4, C_G4_/C_Au_ = 0.5, KCl 50 mM, NaCac 2.5 mM, pH = 6.5.

The effect upon metal complexes binding on the thermal denaturation profile of CT-DNA was studied by recording absorbance data at increasing temperatures for DNA alone, and for mixtures with a 1:1 metal complex/DNA ratio (Figure 4A).

Low ionic strength conditions were used to lower DNA melting temperature: T_m_ ≈ 80 °C in NaCl 0.1 M + NaCac 2.5 mM whereas T_m_ = 61 °C in NaCac 2.5 mM. This latter condition enables us to observe strong stabilisation effects. Indeed, the investigated carbenes induce a strong DNA stabilisation: ∆T_m_ = 20 °C for the [Au(EIA)_2_]^+^/CT-DNA system, but even much higher for the silver complex where the curve does not reach a plateau even at 95 °C.

#### 2.1.3. Viscosity

Equation (3) was used to calculate the relative viscosity.

(3) $$\frac{\eta }{\eta {}^{\circ}\;}=\;\frac{\left(t-t_{\mathrm{solv}}\right)}{\left(t_{\mathrm{DNA}}-t_{\mathrm{solv}}\right)}$$

**Figure 5:**
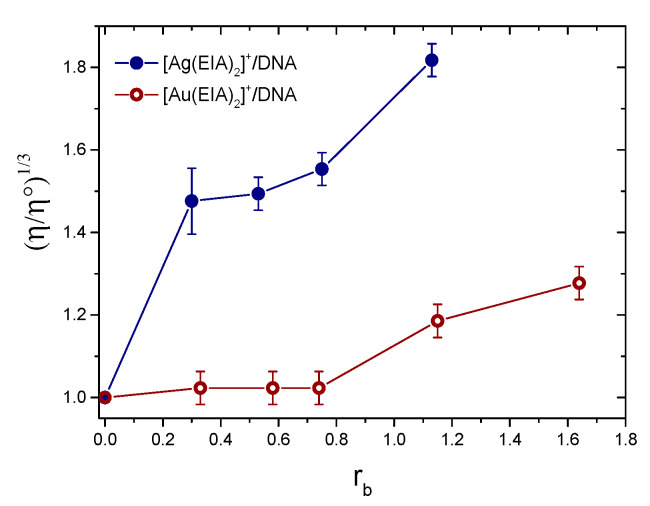
Relative viscosity of DNA mixtures with [Ag(EIA)_2_]^+^ or [Au(EIA)_2_]^+^, r_b_ = [complex]/[DNA], C_DNA_ = 2.10 × 10^−^^5^ M, NaCl 0.1 M, 2.5 mM NaCac, pH = 7.0, T = 25.0 °C.

Here, t_solv_ is the run-off time in seconds for the buffer; t_DNA_ is the run-off time in seconds for CT-DNA solution in the buffer (C_DNA_ = 2.10 × 10^−^^5^ M expressed in base pairs); t is run-off time in seconds for DNA solutions in the presence of an increasing amount of [Ag(EIA)_2_]^+^ or [Au(EIA)_2_]^+^. Following the correlation between viscosity and polymer length [52], we plotted the cubic root of the viscosity vs the concentration ratio r_b_ = C_Ag_/C_DNA_ or C_Au_/C_DNA_ to obtain the graph in Figure 5.

Measurements show that for the silver complex there is a significant increase in viscosity as the [complex]/[DNA] ratio increases, in line with a strong intercalative interaction that modifies the elongation of the DNA strand. Concerning the gold complex, a lower viscosity increase is present. This indicates that the interaction between the gold complex and CT-DNA changes the elongation of the filament to a much lower extent than the silver one. Note that, under experimental conditions close to those used in the present viscometric experiments, the relative viscosity reaches values in the 1.6–2.0 range for Ethidium/DNA mixtures (DNA in base pairs) with ratios r_b_ = 1.0 to 2.3 [53,54,55]. This confirms that the values obtained for the [Ag(EIA)_2_]^+^ complex are in line with those of a known intercalator.

On the whole, as for CT-DNA binding, it might be concluded that the behaviour of the metal complexes results from a complex interplay between external/groove binding of auto-aggregates species and intercalation. In particular, for the [Au(EIA)_2_]^+^/CT-DNA system external/groove binding occurs, with some contribution of a polynucleotide-driven cooperative dye-dye interaction in the presence of dye excess. The binding features are anyway due to non-covalent binding modes, as mass spectrometry experiments done on a mixture of [Au(EIA)_2_]^+^ and DNA oligomers did not evidence the formation of any adduct (not shown). For the [Ag(EIA)_2_]^+^/CT-DNA system the binding mode is still non-covalent but different from the gold compound: for silver intercalation plays a major role (fluorescence data would refer quite only to this binding mode) with some contribution of groove binding (absorbance data are a mixture of the two binding modes). Note that this latter picture is demonstrated not only by the dependence of equilibrium on the EtOH content but also, by inspecting Table 1, we might see that the enthalpy-driven process observed with fluorescence, gradually fades when moving to absorbance towards an entropy-favoured mode. The anthracenyl monocarbenes previously studied by some of us [40] showed comparable binding features towards CT-DNA: the monocationic silver complex intercalates into DNA double helix, while for the neutral gold complex, a groove binding is supposed. Here, the presence of two anthracene intercalating moieties produces a similar but more complex mechanism of action. The dye-dye interactions in solution seem to be disfavoured (see the well-defined peaks/transitions in Figure 2A,B), whereas aggregation on the polymer surface is enhanced (descending/biphasic Figure 2C,D).

### 2.2. Interaction with Synthetic RNAs

The synthetic polynucleotides poly(A), poly(A)poly(U) and poly(A)2poly(U) helices were employed as representative models for single-, double- and triple-stranded RNAs. In absorbance titrations, the silver complex did not produce any change in the poly(A) or poly(A)2poly(U) spectrum (Figure S4). On the other hand, the titration with the poly(A)poly(U) shows the appearance of definite isosbestic points and a slight absorbance decrease (Figure S5). For the gold complex, the addition of each of the RNAs causes a decrease of the absorbance, similar to that observed in the titrations with CT-DNA (Figure S6). Again, the constant value cannot be calculated because the iterative method does not go to convergence, suggesting a quantitative reaction. It is therefore evidenced that the gold complex unselectively binds outside both DNA and RNAs polymers. Conversely, the silver species is selective towards double helices only (drive for intercalation), with much lower affinity than that for CT-DNA (K_poly(A)poly(U)_ = (1.5 ± 3) × 10^3^ M^−^^1^ at NaCl 0.1 M, NaCac 2.5 mM, pH = 7.0, 25.0 °C).

### 2.3. Interaction with DNA G-Quadruplexes (G4s)

As for absorbance titrations, the addition of a DNA telomeric G4 to a solution of [Ag(EIA)_2_]^+^ produced very slight spectral variations indicating a weak interaction with this complex (Figure S7A). Again, for the gold complex, the absorbance behaviour is the same as the one found for the systems already described above (Figure S7C). Thermal denaturation experiments (at λ = 290 nm, Figure 4B) yielded T_m_(G4) = 60 °C, T_m_([Ag(EIA)_2_]^+^/G4) = 60 °C, T_m_([Au(EIA)_2_]^+^/G4) = 57 °C. Therefore, no significant stabilization of the quadruplex is observed; the interaction of the gold carbene even caused a slight destabilization of the G-quadruplex structure. It seems, therefore, that the gold complex again shows unselective outer binding. The silver dicarbene is not prone both to undergo some sitting-atop interaction with the G4 and to intercalate between tetrads. Under these circumstances, it loses its affinity for the biosubstrate.

### 2.4. Interaction with Bovine Serum Albumin (BSA) Protein

#### 2.4.1. Fluorescence Titrations

**Figure 6:**
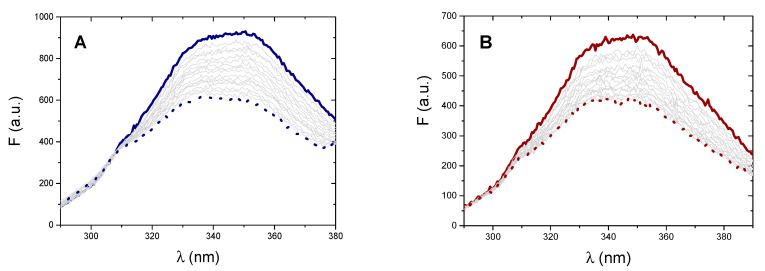
(**A**) Fluorescence spectra of BSA 3.14 × 10^−^^7^ M alone (full line) and (**A**) in the presence of increasing amounts of [Ag(EIA)_2_]^+^, from 0 M to 2.37 × 10^−6^ M; (**B**) in the presence of increasing amounts of [Au(EIA)_2_]^+^, from 0 M to 3.10 × 10^−6^ M (dotted line). λ_ex_ = 280 nm, NaCl 0.1 M, NaCac 2.5 mM, pH = 7.0, T = 27 °C.

Fluorescence experiments were performed at different temperatures (15–50 °C range) by recording the emission spectrum of BSA at increasing amounts of the metal complex (Figure 6). BSA intrinsic fluorescence is excited as λ_ex_ = 280 nm with maximum emission centered at around 350 nm. However, both metal complexes do emit light when excited at 280 nm with an emission band that starts from about 380 nm. To limit any possible interference, the binding isotherms are analysed at λ_em_ = 330 nm.

To verify that the emission decrease was due to adduct formation and not to collisional quenching only, the data were treated with the modified Stern Volmer equation (Equation (4)) [56], which provides the Stern–Volmer constant K_SV_ and f_a_, the fraction BSA fluorophores accessible to the quencher (Q).

(4) $$\frac{F{}^{\circ}}{\Delta F}=\frac{1}{f_{a}K_{\mathrm{SV}}\left[Q\right]}+\frac{1}{f_{a}}$$

Here, F° is the BSA fluorescence in the absence of a metal complex, ΔF = F_BSA_ − (ϕ_BSA_C_BSA_), [Q] is the analytical concentration of the (free) quencher (the metal complex, which is in excess so that free may be approximated with the total). Fluorescence was corrected for inner-filter effects as F_corr_ = F_obs_ × antilog(A_λex_ + A_λem_)/2 where A_λex_ and A_λem_ represent the absorbances at the excitation (280 nm) and emission (330 nm) wavelengths. Table S1 collects the results. For both complexes, the high values of K_SV_ and the absence of K_SV_ increase with temperature indicate adduct formation. The equilibrium constants were obtained using Equation (5), which is based on a 1:1 model.

(5) $$\frac{C_{\mathrm{BSA}}}{\Delta F}=\frac{1}{K\Delta \varphi }\frac{1}{\left[Q\right]}+\frac{1}{\Delta \varphi }$$

**Figure 7:**
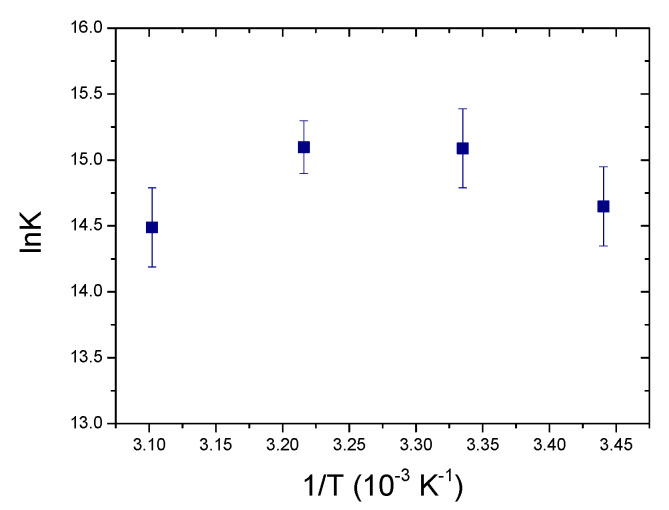
Spectrofluorimetric Van’t Hoff plot for the [Ag(EIA)_2_]^+^/BSA system; NaCl 0.1 M, NaCac 2.5 mM, pH = 7.0.

Here, C_BSA_ is the total analytical concentration of the protein. The numerical evaluation holds for the [Ag(EIA)_2_]^+^/BSA system only (for gold again no convergence) and the relevant data are shown in Figure 7. Within the error, there is no definite trend. This means that ΔH is close to zero while ΔS is positive. These values denote that the interaction occurred is of mere electrostatic nature [57,58,59].

Equation (6), related to the Scatchard equation, may also be applied to the titration data collected for the [Ag(EIA)_2_]^+^/BSA and [Au(EIA)_2_]^+^/BSA systems (C_M_ is the total analytical concentration of the metal compound):

(6) $$\frac{C_{\mathrm{BSA}}{(C}_{M}\Delta \varphi -\Delta F)}{\Delta F}=\frac{1}{\mathrm{nK}’}+\frac{{(C}_{M}\Delta \varphi -\Delta F)}{\Delta \varphi }\frac{1}{n}$$

If the evaluation of K’ is here made impossible by intercepts too close to zero, this analysis yields a binding stoichiometry (n) of 1.0 ± 0.1 for both systems (Figure S8). This confirms the suitability of Equation (5) to calculate the equilibrium constants.

#### 2.4.2. Synchronous Fluorescence Spectra

Synchronous fluorescence spectroscopy is used to study the interaction between metal complexes and BSA; in fact, synchronous fluorescence spectra at Δλ = 60 nm focus on BSA tryptophan residues (Trp) only, whereas Δλ = 15 nm on tyrosine residues (Tyr) only [60]. The shift in the position of the maximum emission would correspond to changes in polarity around the chromophore molecule. For both complexes and in both cases (Figures S9 and S10), there are no changes in the maximum emission wavelength. According to the literature, the lack of spectral shifts indicates the absence of direct short-range interaction with Trp or Tyr residues. On the other hand, the significant decrease in the intensity of the Trp emission (quite absent for Tyr and particularly significant in the case of gold) may be due to a long-range interaction. BSA contains two tryptophan residues: Trp-134, located on the surface of domain IA, and Trp-212, embedded within the hydrophobic pocket of domain IIB. The Cys-34 residue is located quite close to Trp-134.

#### 2.4.3. Mass Spectrometry

**Figure 8:**
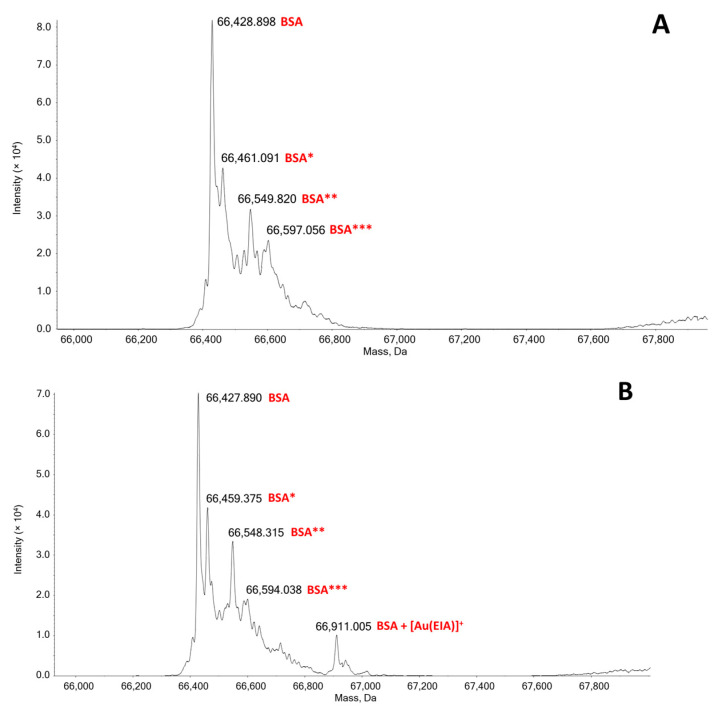
Deconvoluted ESI mass spectra of BSA 5.50 × 10^−7^ M (**A**) and [Au(EIA)_2_]^+^/BSA (**B**) in a protein:metal ratio = 1:1.5 in 1 × 10^−4^ M ammonium acetate solution at pH 6.8 and recorded after 48 h of incubation at 37 °C. BSA* = sulfinilation on Cys-34; BSA** = cysteinylation on Cys34; BSA*** = glycosylated form of BSA.

High-resolution ESI-MS experiments were performed first by recording the mass spectrum of BSA alone. In this spectrum, shown in Figure 8A, the main peak at 66,428 Da belongs to the BSA. Moreover, some other small peaks are also present at 66,461, 66,549, and 66,597 Da corresponding to a few post-translational modifications of the protein [30]. Then, ESI mass spectra were acquired for BSA after 0, 24 and 48 h of incubation at 37 °C with the silver complex in 1:1, 1.5:1 and 3:1 Ag/BSA ratios. For the MS analysis with the gold complex, the spectra were acquired on the protein solution incubated in the same conditions with the gold compound in 1:1, 1.5:1, and 3:1 Au/BSA ratios. None of the spectra of the Ag compound/BSA system shows the formation of an adduct (not shown). This agrees with the weak electrostatic binding evidenced by fluorometric titrations. On the other hand, samples containing the gold complex showed the presence of a new peak at around 66,911 Da, attributable to an adduct between BSA and the gold complex that has lost one of the two NHC moieties (Figure 8). The covalent binding is reasonably supposed to take place on the Cys-34 residue as already described in the literature for other gold complexes [30,34,35]. This result also agrees with that established by fluorescence titrations, where high values of equilibrium constants, characteristic of covalent bonds formation, were found. Note that the rate of Au(I)/cysteine adduct formation is known to be fast [61,62] and that signal stabilisation was carefully checked during the titrations. The intensity of the peak increases with the amount of complex in solution, but no time evolution of the spectra was observed. Also, binding to Cys-34 may have some effect on Trp-134 emission, in agreement with synchronous spectra results.

On the whole, as for BSA binding, it might be concluded that the silver complex interacts with the protein but with a reversible weak process based on electrostatics. Oppositely, as for gold, both synchronous spectra and ESI-MS experiments concur in evidencing the presence of a stronger ligand exchange reaction with the formation of a covalent protein adduct.

## 3. Materials and Methods

### 3.1. Materials

The silver dicarbene [Ag(EIA)_2_]Cl was prepared according to the procedure reported in [41]. Subsequently, for the synthesis of [Au(EIA)_2_]Cl, 22.2 mg of [Ag(EIA)_2_]Cl were suspended in 3 mL of dichloromethane together with 10 mg of chloro(dimethyl sulfide)gold(I). The mixture was stirred at room temperature for 2 h, then the formed AgCl was removed through filtration. The final product was precipitated through the addition of hexane and recovered by filtration (13.2 mg). Yield 52%. Elemental analysis of C, H, and N for C_40_H_36_N_4_AuCl: calculated C: 59.67%, H: 4.51%, N: 6.96%; experimental: C: 58.98%, H: 4.40%, N: 7.19%. HR-ESI-MS for C_40_H_36_N_4_Au: measured *m*/*z* = 769.25852; theoretical *m*/*z* = 769.26001; mass error = −1.9 ppm (Figure S11). ^1^H-NMR (400.13 MHz, MeOD) δ: 8.59 (s, 2H, Ant H10), 8.18 (d, 4H, Ant H1, *J* = 8.1 Hz), 8.07 (d, 4H, Ant H4, *J* = 8.2 Hz), 7.53–7.45 (m, 8H, Ant H2,3), 7.31 (s, 2H, Im H4′), 7.23 (s, 2H, Im H5′), 5.91 (s, 4H, AntCH_2_Im), 3.58 (b, 4H, ImCH_2_CH_3_), 1.13 (b, 6H, ImCH_2_CH_3_).

Calf thymus DNA (CT-DNA in the text), natural double-stranded B-DNA, was purchased from Sigma-Aldrich. Its solutions were prepared by dissolving the solid in ultrapure water under mild mixing conditions for 24 h. The solution was sonicated to obtain about 500 base-pair long polynucleotides (gel agarose test). The concentration in the prepared stock solution was approximately 2.5 mM (expressed in base pairs) in ultra-pure water. Before its use, the exact concentration value (C_DNA_, in base pairs) was measured considering ε_260_ = 13,200 cm^−1^M^−1^ [63]. Poly(A) and poly(U) were purchased from Sigma-Aldrich as model polynucleotides for RNA structures. The two stock solutions were prepared by dissolving a known quantity of the solid polynucleotide in the buffer NaCl 0.1 M, NaCac 2.5 mM, pH = 7.0. The concentrations of single strands were spectrophotometrically evaluated (ε = 10110 cm^−1^M^−1^ at 257 nm [64]) and ε = 8900 cm^−1^M^−1^ at 260 nm [64] respectively for a concentration in phosphate groups, C_A_ and C_U_. The concentration of the stock solutions was 8.4 mM for poly(A) and 14 mM for poly(U). To form synthetic RNA double helix, poly(A) and poly(U) were mixed 1:1 in buffer (NaCl 0.1 M, NaCac 2.5 mM, pH = 7.0) and let equilibrate overnight in the dark at room temperature to get the double-stranded poly(A)poly(U) (C_AU_ = 2.0 mM in base pairs) [65]. Similarly, poly(A)poly(U) and poly(U) were mixed 1:1 in the same buffer and left overnight at room temperature to obtain the RNA triple helix (C_A2U_ = 4.0 × 10^−4^ M in base triplets) [66]. A telomeric G-quadruplex structure was prepared by dissolving a 23-residue human telomer (Tel-23: 5′-TAG GGT TAG GGT TAG GGT TAG GG-3′, Sigma-Aldrich, St. Louis, MO, USA) in 50 mM KCl, 2.5 mM NaCac, pH = 6.5. The solution was slowly heated up to 90–95 °C, maintained for 10–15 min and then slowly cooled down in the water bath and left overnight; a 1 mM stock solution (C_G4_, in strands) was obtained [67]. The BSA solution was prepared by weighing lyophilized BSA purchased from Sigma-Aldrich (PM ≈ 66 kDa), and by dissolving it in NaCac 2.5 mM with NaCl 0.1 M at pH = 7.0. The effective concentration was determined spectrophotometrically considering the absorption at 278 nm (ε = 45,000 cm^−1^M^−1^ [68]). The stock solution was C_BSA_ = 8.0 × 10^−5^ M.

### 3.2. Methods

The pH measurements were performed with a “Ω Metrohm 713 pH Meter” (Filderstadt, Germany). All the buffers at pH 7.0 were prepared by dissolving the suitable amount of salt (NaCl, KCl and sodium cacodylate NaCac) in ultra-pure water. In case of need, the pH was corrected with micro additions of concentrated HCl or NaOH. Ultra-pure water was obtained through an “AriumPro SARTORIUS” apparatus (Göttingen, Germany). The stock solutions of the metal complexes were prepared in DMSO at concentrations of about 2.0 mM and then diluted in a suitable buffer to obtain the solutions for the analysis. In the working solutions, the dilution was such that the quantity of DMSO could be considered negligible (maximum 1–2% and well below 1% in the fluorescence and mass experiments). A UV-2450 SHIMADZU double-beam UV-vis spectrophotometer (Kyoto, Japan) was used for absorbance, while fluorescence was recorded with a LS55 Perkin-Elmer spectrofluorometer (Waltham, MA, USA). Both instruments have jacketed cell holders providing temperature control within 0.1 °C. All spectra were recorded using quartz cuvettes of 1000 µL or 500 µL volume and an optical path of 1 cm. For the titrations, increasing quantities of the titrating solution were added to the sample thanks to a glass micro-syringe Hamilton (Reno, NV, USA) equipped with a Mitutoyo micro-screw (1 whole turn = 8.2 µL). Absorbance and fluorescence titrations were carried out by adding increasing amounts of the nucleotide solution to a solution of the metal complex in the buffer (NaCl 0.1 M, NaCac 2.5 mM, pH = 7.0). All the spectra shown are corrected for dilution factors. Fluorescence experiments conditions were carefully chosen (high dilution, wavelengths) and checked so to ensure direct proportionality between reading and concentration. Absorption titrations at different ethanol concentrations were carried out by adding the suitable volume percentage of EtOH in the buffer. The thermal denaturation curves of samples containing the investigated complex and polynucleotide in equimolar amounts were measured using absorbance changes (260 nm for CT-DNA and 290 nm for G-quadruplex) at increasing temperatures ranging from ~25 °C to ~95 °C. The percentage of absorbance change (%A change = 100 × (A(T) − A°)/(A∞ − A°)), where A(T) is the absorbance read at each temperature T (°C), A° the absorbance corresponding to the initial plateau and A∞ the absorbance for the final plateau, was plotted against temperature. In this way, a sigmoidal curve could be obtained. The melting temperature was derived as the maximum of the first derivative of the sigmoidal curve. All the experiments were performed under low ionic strength conditions (buffer NaCl 0 M, NaCac 2.5 mM, pH = 7.0). The viscosimetric analyses were performed with a semi-micro “Cannon-Ubbelohde” capillary viscometer (State College, PA, USA): solutions containing increasing dye/DNA molar ratios were prepared in NaCac 2.5 mM with NaCl 0.1 M at pH = 7.0. During the experiments, the temperature was kept constant at 25.0 °C employing a thermostatic water bath. For the fluorescence titrations of BSA with the two metal complexes, an increasing amount of the metal complex solution was added to a solution of BSA 3.14 × 10^−7^ M in NaCac 2.5 mM with NaCl 0.1 M at pH = 7.0 and λ_exc_ = 280 nm.

For mass spectrometry, we used an AB SCIEX Triple TOF 5600^+^ high-resolution mass spectrometer (Sciex, Framingham, MA, USA), equipped with a DuoSpray^®^ interface operating with an ESI probe. Respective ESI mass spectra were acquired through direct infusion at 7 μL min^−1^ flow rate. The samples were prepared in LC-MS grade solvents, following a well-established protocol previously developed [29,30,35,69]. To promote the ionization in positive mode, 0.1% *v*/*v* of formic acid was added just before infusion. The BSA solutions were prepared in 10 mM ammonium acetate solution (pH 6.8) and finally diluted in LC-MS grade H_2_O obtaining a final BSA concentration of about 5 × 10^−7^ M. BSA/metal complexes mixtures were prepared following the same procedure. The ESI source parameters were optimized and were as follows: positive polarity, ion spray voltage floating 5500 V, temperature 25 °C, ion source gas 1 (GS1) 45 L min^−1^; ion source gas 2 (GS2) 0; curtain gas (CUR) 12 L min^−1^, declustering potential (DP) 150 V, collision energy (CE) 10 V, acquisition range 1000–2600 *m*/*z*. For acquisition, Analyst TF software 1.7.1 (Sciex, Framingham, MA, USA) was used and deconvoluted spectra were obtained by using the Bio Tool Kit micro-application v.2.2 embedded in PeakView^TM^ software v.2.2 (Sciex).

## 4. Conclusions

Herein, we have investigated the interaction of a new silver(I) bis-NHC anthracenyl complex and its gold(I) analogue with a series of biosubstrates.

[Ag(EIA)_2_]Cl exhibited a strong affinity and selectivity for the double helix structure of polynucleotides, with a greater affinity for CT-DNA compared to RNA. Oppositely, no interaction towards single or triple RNA helices nor with DNA G-quadruplexes was observed. Spectroscopic titrations, viscometry and thermal denaturation experiments indicate that the silver carbene can intercalate between the base pairs of the double helix, but an interaction with the groove also may take place depending on the experimental conditions. As for the BSA protein mass spectra and titrations with BSA suggest low affinity: the complex interacts with this model protein through a non-covalent bond according to a weak electrostatically-driven process (hydrophobicity does not seem to play a significant role even if [Ag(EIA)_2_]^+^ is bearing two hydrophobic anthracenes).

At variance, the behaviour of the gold compound is the opposite. [Au(EIA)_2_]^+^ unselectively interacts with all DNA and RNA biosubstrates. An external interaction with a strong electrostatic contribution occurs between the polynucleotide phosphates and the positively charged metal complex. It is expected that this type of interaction does not produce changes in the biochemical properties of the nucleic acids. Despite the presence of the two anthracenyl residues (which may drive intercalation), this is not observed for [Au(EIA)_2_]^+^, neither are some covalent adducts with polynucleotides. As for proteins, the picture changes. Fluorescence titrations and mass spectrometry demonstrated the formation of a covalent adduct with BSA after one NHC ligand is lost. Given previous works [70,71], it is legitimate to assume that Cys-34 is involved in the binding.

These results describe the very different reactivity features for the two complexes and enlighten the crucial role played by the metal centre to selectively drive the reactivity towards a precise biosubstrate.

## Figures and Tables

**Table 1:** Equilibrium constant (K) at different temperatures and thermodynamic parameters obtained from spectrophotometric and spectrofluorimetric titrations for the [Ag(EIA)_2_]^+^/CT-DNA system; NaCl 0.1 M, NaCac 2.5 mM, pH = 7.0.

T (°C)	K_ABS_ (10^3^ M^−1^)	K_FLUO_ (10^3^ M^−1^)
15.0	10 ± 1	27 ± 2
25.0	7.4 ± 0.2	20 ± 3
35.0	5.1 ± 0.3	10 ± 2
48.0	3.7 ± 0.4	5.4 ± 0.3
ΔH (kJ/mol)	−23.5 ± 0.3	−37.9 ± 0.9
ΔS (J/molK)	−5 ± 3	−46 ± 3
−TΔS (kJ/mol)	2 ± 1	14 ± 1

## Supplementary Materials

The following are available online. Binding isotherms for [Ag(EIA)_2_]Cl/CT-DNA and [Au(EIA)_2_]Cl /CT-DNA (absorbance titrations and fluorescence titrations); example of Scatchard plot for Ag(EIA)_2_Cl/CT-DNA (absorbance titrations); absorption spectra and relevant binding isotherms for the [Ag(EIA)_2_]Cl/poly(A), [Ag(EIA)_2_]Cl/poly(A)poly(U), [Ag(EIA)_2_]Cl/poly(A)2poly(U), [Au(EIA)_2_]Cl/poly(A), [Au(EIA)_2_]Cl/poly(A)poly(U) and [Au(EIA)_2_]Cl/poly(A)2poly(U) systems; absorption spectra and relevant binding isotherms for [Ag(EIA)_2_]Cl/G4 and [Au(EIA)_2_]Cl/G4 systems; example of application of Equation (6) on [Ag(EIA)_2_]Cl/BSA and [Au(EIA)_2_]Cl/BSA systems; synchronous fluorescence spectra on [Ag(EIA)_2_]Cl/BSA and [Au(EIA)_2_]Cl/BSA systems at Δλ = 60 nm and Δλ = 15 nm; high-resolution ESI mass spectrum of [Au(EIA)_2_]^+^.
