# Supplementary material for: On the Different Mode of Action of Au(I)/Ag(I)-NHC Bis-Anthracenyl Complexes Towards Selected Target Biomolecules
Source: Molecules. 2020 Nov 20;25(22):5446. doi: 10.3390/molecules25225446 (PMC7699860; doi:10.3390/molecules25225446)
Supplement: Supplementary file 1 [file molecules-25-05446-s001.pdf]

SUPPORTING INFORMATION

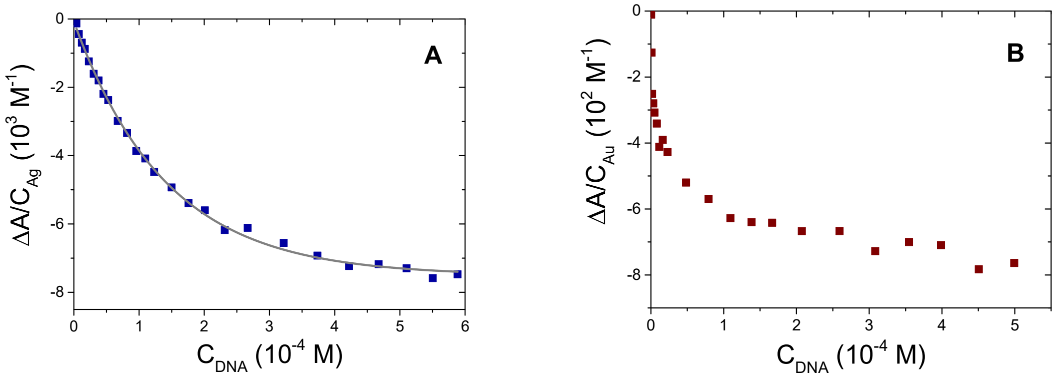

Figure S1. (**A**) Change in absorbance as a function of the DNA concentration for the $[Ag(EIA)_2]^+$/CT-DNA system, C_{Ag} = 7.96 × 10^{-6} M. (**B**) Change in absorbance as a function of the DNA concentration for the $[Au(EIA)_2]^+$/CT-DNA system, C_{Au} = 4.34 × 10^{-5} M. NaCl 0.1 M, NaCac 2.5 mM, pH = 7.0, T = 25.0 ° C.

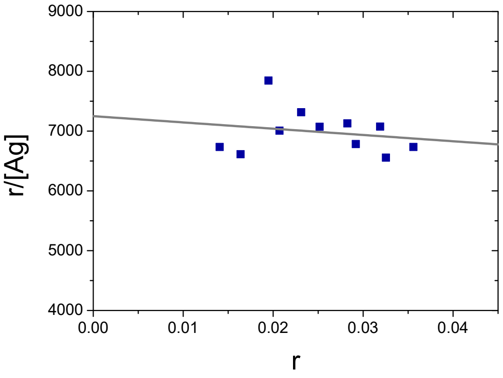

Figure S2. Scatchard plot for the $[Ag(EIA)_2]^+$/CT-DNA system; C_{Ag} = 7.96 × 10^{-6} M, NaCl 0.1 M, NaCac 2.5 mM, pH = 7.0, T = 25.0 ° C

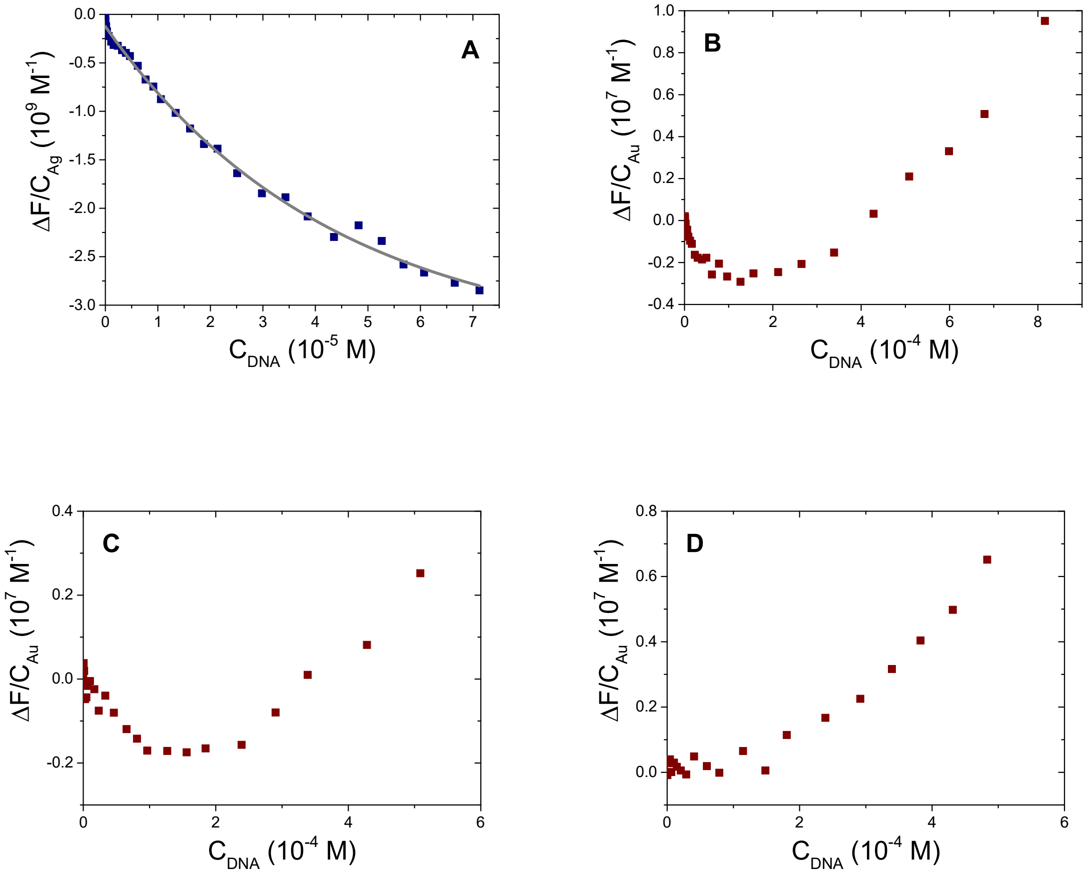

Figure S3. Change in fluorescence as a function of the CT-DNA concentration: for the $[Ag(EIA)_2]^+$/CT-DNA system, C_{Ag} = 7.86 × 10^{-8} M, NaCl 0.1 M, NaCac 2.5 mM, pH = 7.0, T = 25.0 ° C (**A**); for the $[Au(EIA)_2]^+$/CT-DNA system, C_{Au} = 7.71 × 10^{-6} M (**B**) NaCl 0.1 M, NaCac 2.5 mM, pH = 7.0, T = 25.0 ° C, (**C**) NaCl 0.1 M, NaCac 2.5 mM, pH = 7.0, T = 37.0 ° C; (**D**) NaCl 1.0 M, NaCac 2.5 mM, pH = 7.0, T = 25.0 ° C.

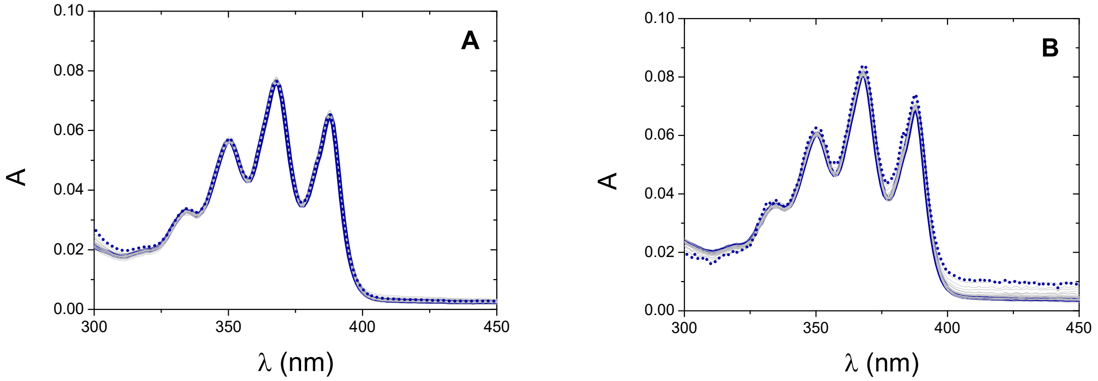

Figure S4. (**A**) Absorption spectra of $[Ag(EIA)_2]^+$ 7.96 × 10^{-6} M alone (—) and in the presence of increasing amounts of poly(A), from 0 M to 2.34 × 10^{-4} M (…….), spectra are corrected for the dilution factor. (**B**) Absorption spectra of $[Ag(EIA)_2]^+$ 7.96 × 10^{-6} M alone (—) and in the presence of increasing amounts of poly(A)2poly(U), from 0 M to 1.67 × 10^{-4} M (…….), spectra are corrected for the dilution factor.

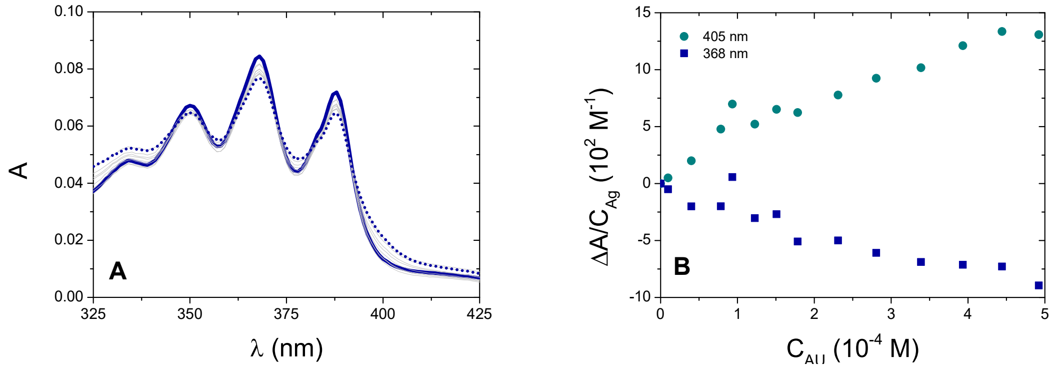

Figure S5. (**a**) Absorption spectra of $[Ag(EIA)_2]^+$ 7.96 × 10^{-6} M alone (—) and in the presence of increasing amounts of poly(A)poly(U), from 0 M to 4.92 × 10^{-4} M (…….), spectra are corrected for the dilution factor and (**b**) relevant change in absorbance as a function of the poly(A)poly(U) concentration. NaCl 0.1 M, NaCac 2.5 mM, pH = 7.0, T = 25.0 ° C.

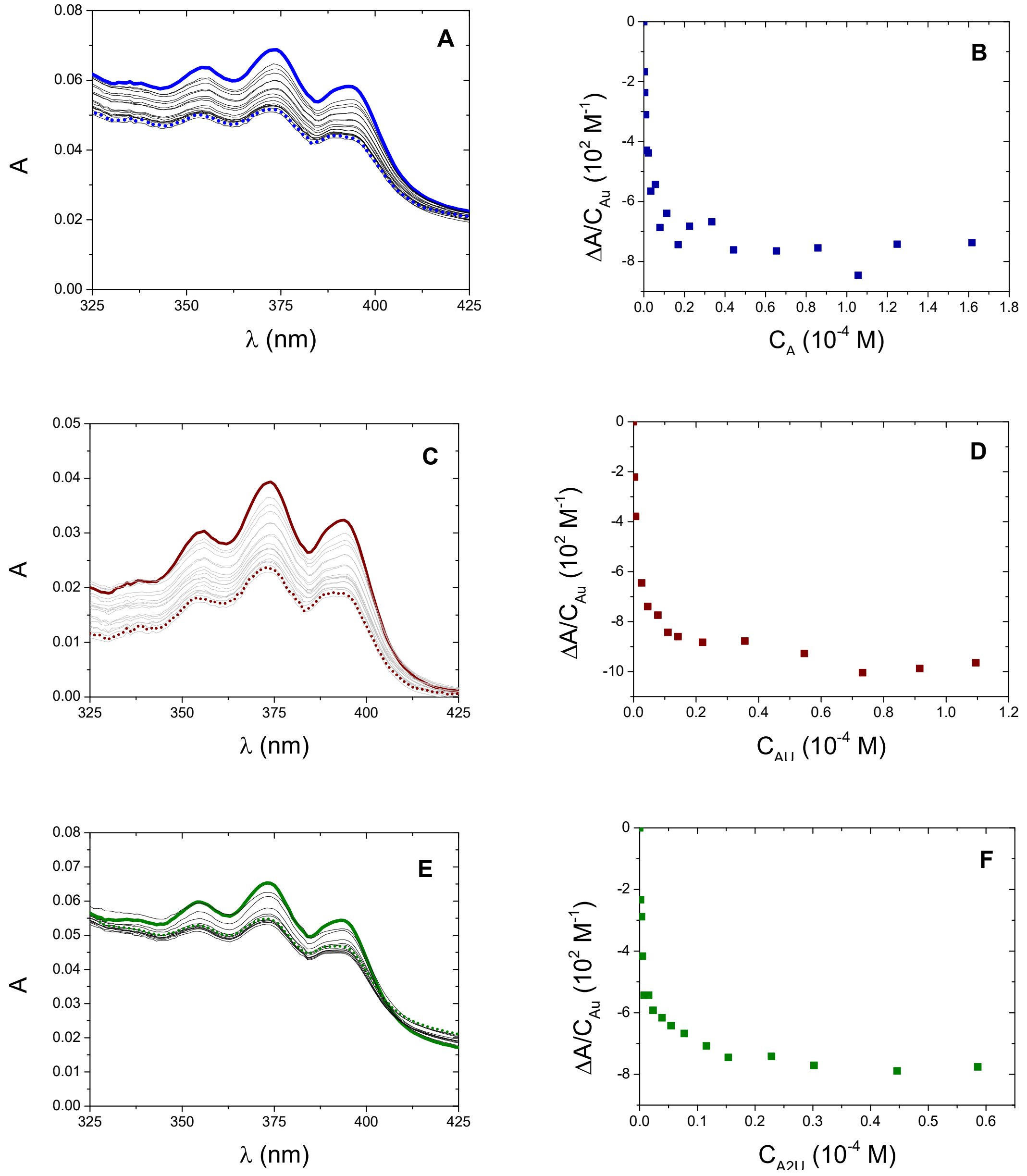

Figure S6. (**A**) Absorption spectra of $[Au(EIA)_2]^+$ 1.74 × 10^{-5} M alone (—) and in the presence of increasing amounts of poly(A), from 0 M to 1.96 × 10^{-4} M (…….) and (**B**) relevant change in absorbance. (**C**) Absorption spectra of $[Au(EIA)_2]^+$ 1.74 × 10^{-5} M alone (—) and in the presence of increasing amounts of poly(A)poly(U), from 0 M to 1.10 × 10^{-4} M (…….) and (**D**) relevant change in absorbance. (**E**) Absorption spectra of $[Au(EIA)_2]^+$ 1.74 × 10^{-5} M alone (—) and in the presence of increasing amounts of poly(A)2poly(U), from 0 M to 5.86 × 10^{-5} M (…….) and (**F**) relevant change in absorbance. NaCl 0.1 M, NaCac 2.5 mM, pH = 7.0, T = 25.0 ° C.

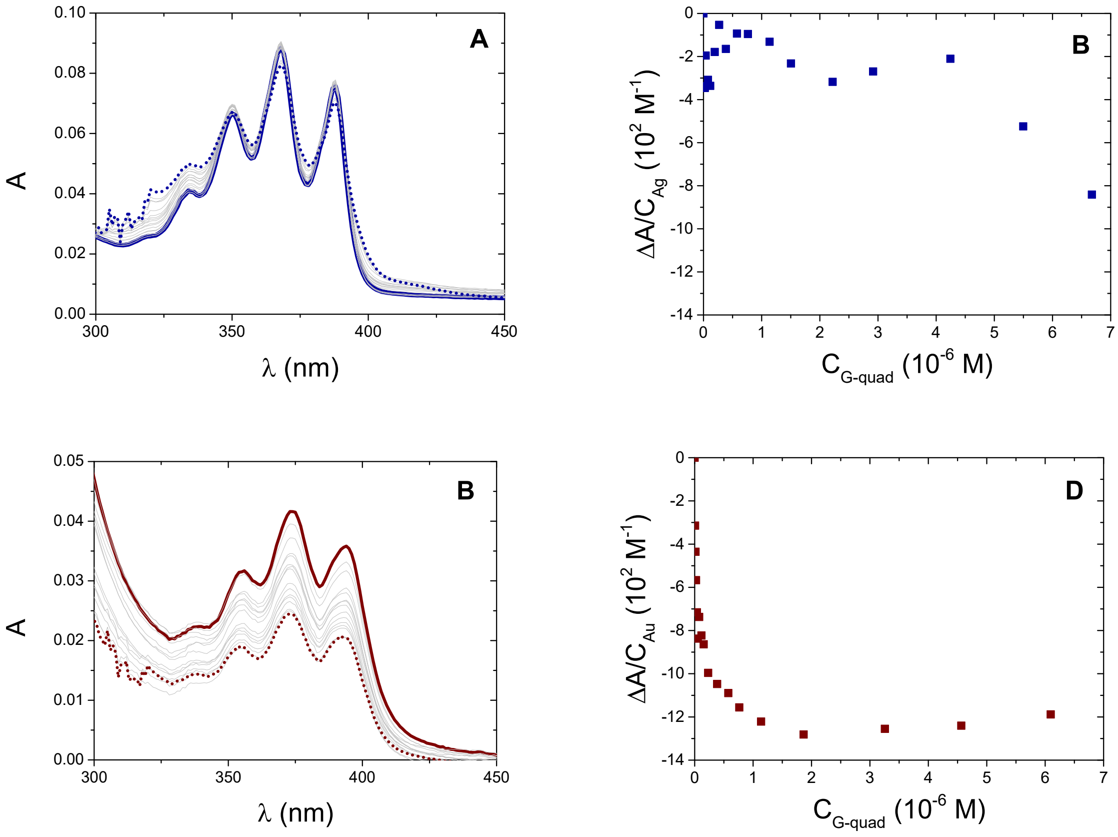

Figure S7. (**A**) Absorption spectra of $[Ag(EIA)_2]^+$ 7.96 × 10^{-6} M alone (—) and in the presence of increasing amounts of G4, from 0 M to 6.68 × 10^{-6} M (…….) and (**B**) relevant change in absorbance. (**C**) Absorption spectra of $[Au(EIA)_2]^+$ 1.74 × 10^{-5} M alone (—) and in the presence of increasing amounts of G4, from 0 M to 6.10 × 10^{-6} M (…….) and (**D**) relevant change in absorbance. KCl 50 mM, NaCac 2.5 mM, pH = 6.5, T = 25.0 ° C.

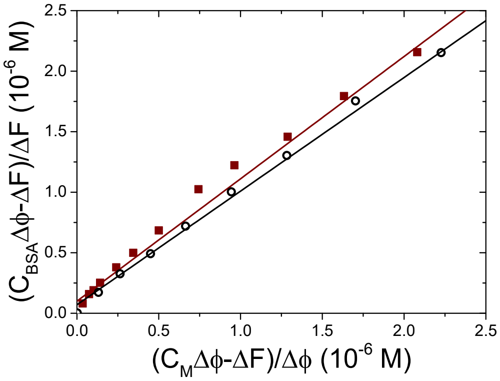

Figure S8. Example of analysis, according to Equation (8), of the data collected by fluorescence titrations on the $[Ag(EIA)_2]^+$/BSA (full square) and $Au(EIA)_2]^+$/BSA (open circle) systems. Experimental conditions are those of Figure 6.

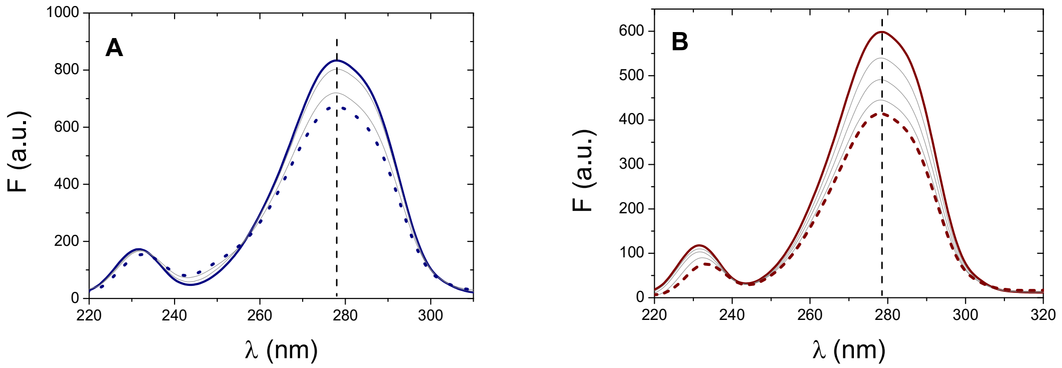

Figure S9. Synchronous fluorescence spectra with $\Delta\lambda$ = 60 nm of BSA 3.14 × 10^{-7} M alone (—) and (**A**) in the presence of increasing quantities of $[Ag(EIA)_2]^+$, from 0 M to 2.37 × 10^{-6} M (**....**), (**B**) in the presence of increasing quantities of $[Au(EIA)_2]^+$, from 0 M to 3.10 × 10^{-6} M (**....**). NaCl 0.1 M NaCac 2.5 mM, pH = 7.0, T = 26.8 °C. All spectra are corrected for dilution.

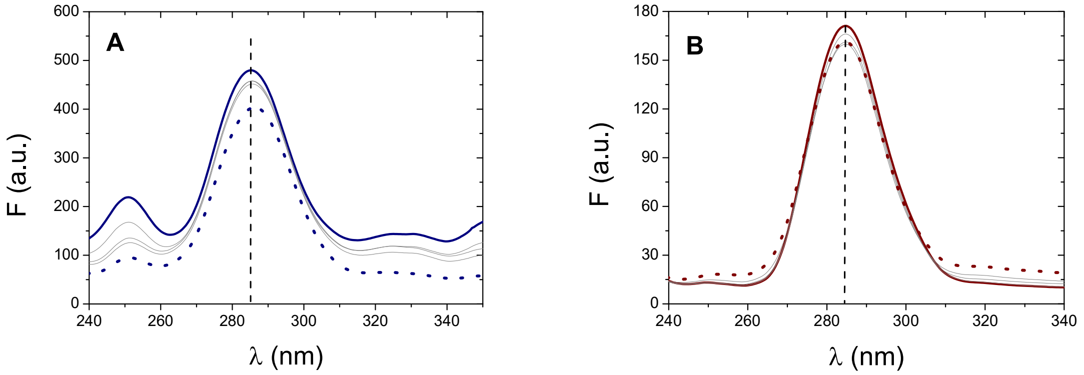

Figure S10. Synchronous fluorescence spectra with $\Delta\lambda$ = 15 nm of BSA 3.14 × 10^{-7} M alone (—) and (**A**) in the presence of increasing quantities of $[Ag(EIA)_2]^+$, from 0 M to 2.37 × 10^{-6} M (**....**), (**B**) in the presence of increasing quantities of $[Au(EIA)_2]^+$, from 0 M to 3.10 × 10^{-6} M (**....**). NaCl 0.1 M NaCac 2.5 mM, pH = 7.0, T = 26.8 °C. All spectra are corrected for dilution.

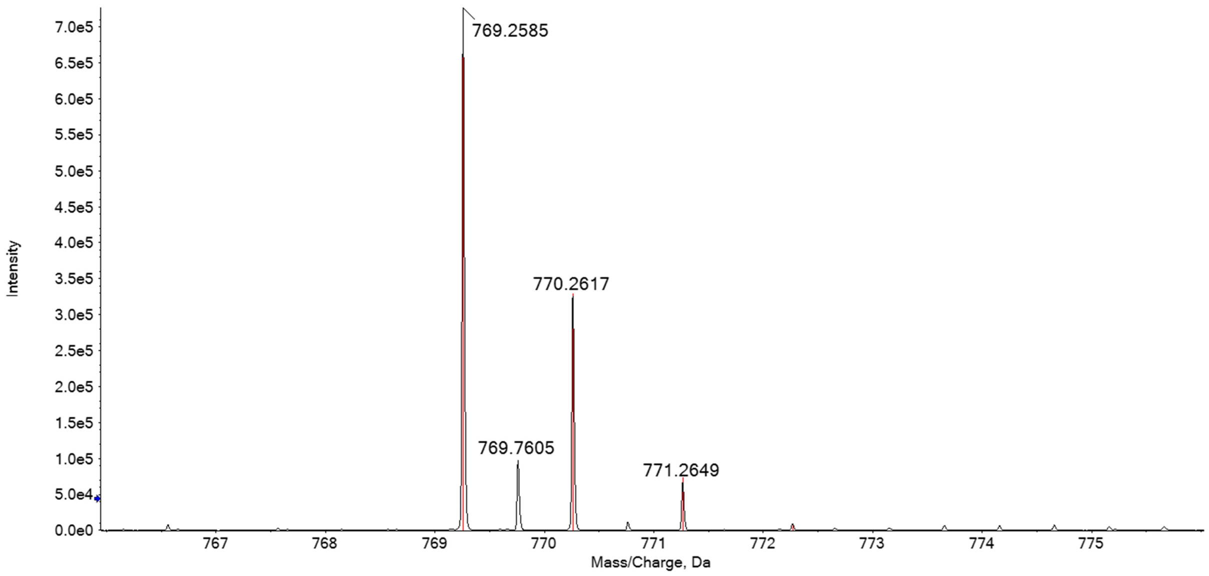

Figure S11. High-resolution ESI mass spectrum of $[Au(EIA)_2]^+$, 10^{-5} M in MeOH. Experimental isotopic distribution for $C_{40}H_{36}AuN_4$ (black line) *vs* the theoretical one (red lines). Measured *m*/*z* = 769.25852; theoretical *m*/*z* = 769.26001; mass error = -1.9 ppm.

Table S1. Stern Volmer constant (K_{SV}) and BSA fraction accessible to the quencher (f_a) at different temperatures obtained by equation (4) by spectrofluorimetric titrations for the BSA / $[Ag(EIA)_2]^+$ and BSA / $[Au(EIA)_2]^+$ systems. Equilibrium constant (K) obtained by equation (5) for BSA / $[Ag(EIA)_2]^+$ system. NaCl 0.1 M, NaCac 2.5 mM, pH = 7.0.

**BSA / $[Ag(EIA)_2]^+$**				**BSA / $[Au(EIA)_2]^+$**		
**T (°C)**	**f_a**	**K_{SV} (10^6 M^{-1})**	**K (10^6 M^{-1})**	**T(°C)**	**f_a**	**K_{SV} (10^6 M^{-1})**
17.5	0.24 ± 0.02	1.7 ± 0.3	2.3 ± 0.6	17.6	0.29 ± 0.03	2.8 ± 0.4
26.7	0.26 ± 0.03	6.3 ± 0.4	4.0 ± 1.0	27.0	0.28 ± 0.07	0.6 ± 0.1
37.8	0.21 ± 0.02	3.5 ± 0.1	3.6 ± 0.7	37.8	0.21 ± 0.01	2.0 ± 0.5
49.2	0.25 ± 0.03	1.2 ± 0.2	2.0 ± 0.5	48.6	0.35 ± 0.01	7.0 ± 0.1
